# Crystal structure and Hirshfeld surface analysis of 1-benzyl-3-(prop-2-yn-1-yl)-2,3-di­hydro-1*H*-1,3-benzo­diazol-2-one

**DOI:** 10.1107/S2056989018016298

**Published:** 2018-11-22

**Authors:** Asmaa Saber, Nada Kheira Sebbar, Tuncer Hökelek, Mohamed El hafi, Joel T. Mague, El Mokhtar Essassi

**Affiliations:** a Laboratoire de Chimie Organique Hétérocyclique URAC 21, Pôle de Compétence Pharmacochimie, Av. Ibn Battouta, BP 1014, Faculté des Sciences, Université Mohammed V, Rabat, Morocco; b Laboratoire de Chimie Bioorganique Appliquée, Faculté des Sciences, Université Ibn Zohr, Agadir, Morocco; c Department of Physics, Hacettepe University, 06800 Beytepe, Ankara, Turkey; d Department of Chemistry, Tulane University, New Orleans, LA 70118, USA

**Keywords:** crystal structure, benzo­diazole, hydrogen bond, alkyne, Hirshfeld surface

## Abstract

The title compound, C_17_H_14_N_2_O, is built up from the planar benzo­diazole unit linked to the benzyl and propynyl substituents. The substituents are rotated significantly out of the benzo­diazole plane, where the benzyl group is inclined by 68.91 (7)° to the benzo­diazole unit. In the crystal, the mol­ecules are linked via inter­molecular C—H_Bnzdzl_⋯O and C—H_Bnzy_⋯O (Bnzdzl = benzo­diazole and Bnzy = benz­yl) hydrogen bonds, enclosing *R*_4_^4^(27) ring motifs, into a network consisting of rectangular layers parallel to the *bc* plane which are also stacked along the *a*-axis direction being associated through C—H⋯π (ring) inter­actions. The Hirshfeld surface analysis of the crystal structure indicates that the most important contributions for the crystal packing are from H⋯H (43.6%), H⋯C/C⋯H (42.0%) and H⋯O/O⋯H (8.9%) inter­actions.

## Abstract 2

In the title compound, the benzo­diazole unit is planar while the benzyl and propynyl substituents are rotated significantly out of this plane.

## Chemical context   

The benzimidazole nucleus constitutes an important pharmacophore in medicinal chemistry and pharmacology (Ouzidan *et al.*, 2011 ▸; Dardouri *et al.*, 2011 ▸; Soderlind *et al.*, 1999 ▸). Benzimidazol-2-one derivatives are of wide inter­est because of their diverse biological activities such as anti­microbial, anti-fungal, anti-histaminic, anti-inflammatory, anti­viral and anti-oxidant (Walia *et al.*, 2011 ▸; Luo *et al.*, 2011 ▸; Ayhan-Kılcıgil *et al.*, 2007 ▸; Navarrete-Vázquez *et al.*, 2001 ▸).
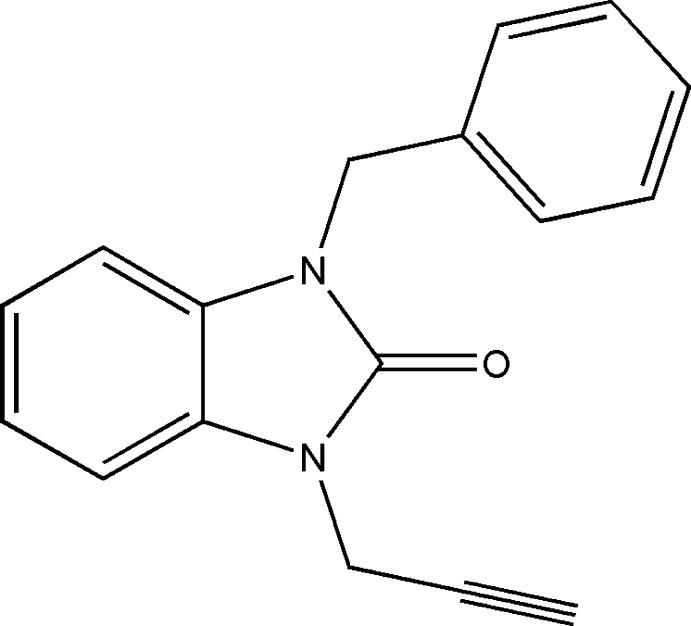


As a continuation of our research works devoted to the development of substituted benzimidazol-2-one derivatives (Lakhrissi *et al.*, 2008 ▸; Mondieig *et al.*, 2013 ▸), we report herein the synthesis, the mol­ecular and crystal structures along with the Hirshfeld surface analysis of a new benzimidazol-2-one derivative, namely 2-benzyl-1-(prop-2-yn­yl)-1*H*-benzoimidazol 2(3*H*)-one. It was obtained by condensation of benzyl chloride with 1-(prop-2-yn­yl)-1*H*-benzoimidazol-2(3*H*)-one in the presence of tetra-*n*-butyl­ammonium bromide as catalyst and potassium carbonate as base.

## Structural commentary   

**Figure 1:**
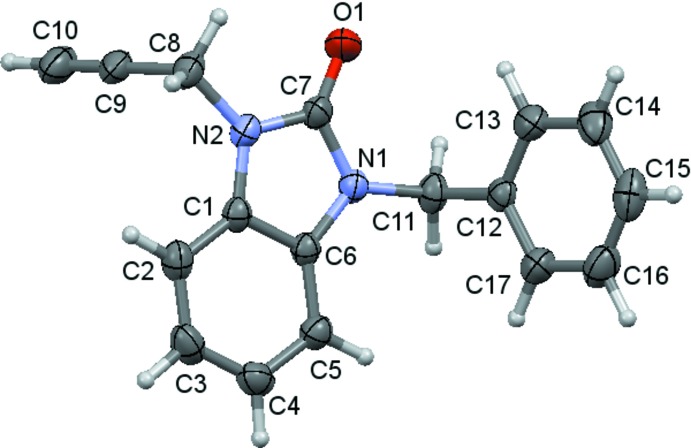
The mol­ecular structure of the title compound with the atom-numbering scheme. Displacement ellipsoids are drawn at the 30% probability level.

The title compound is built up from a benzo­diazole unit linked to benzyl and propynyl substituents (Fig. 1 ▸). The benzo­diazole moiety is planar to within 0.015 (1) Å (for atom C7), and the r.m.s. deviation of the fitted atoms is 0.008 Å. It is inclined by 68.91 (7)° to the C12–C17 ring plane. The benzyl substituent is nearly perpendicular to the benzodizole plane, as indicated by the C6—N1—C11—C12 torsion angle of −87.00 (15)° while the propynyl substituent is at a smaller angle [C1—N2—C8—C9 = −73.46 (18)°]. Atoms O1, C8 and C11 deviate by 0.038 (1), 0.003 (2) and 0.047 (2) Å, respectively, from the benzodizole plane.

## Supra­molecular features   

**Figure 2:**
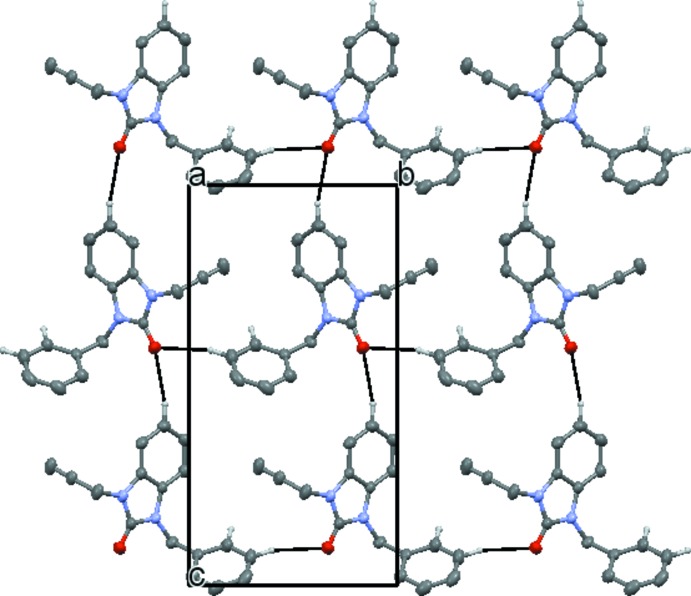
Plan view of a portion of one layer seen along the *a*-axis direction. Inter­molecular C—H_Bnzdzl_⋯O and C—H_Bnzy_⋯O (Bnzdzl = benzo­diazole and Bnzy = benz­yl) hydrogen bonds are shown by dashed lines.

**Figure 3:**
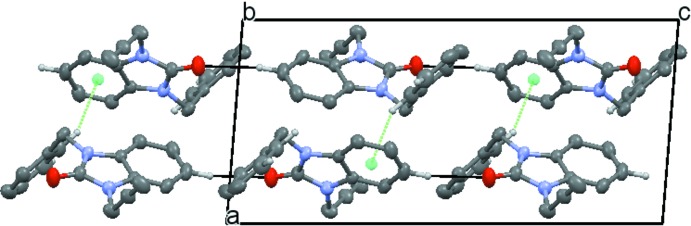
Elevation view of two layers seen along the *b*-axis direction. C—H⋯O hydrogen bonds are shown by black dashed lines while C—H⋯π(ring) inter­actions are shown by green dashed lines.

In the crystal, the mol­ecules are linked via inter­molecular C—H_Bnzdzl_⋯O and C—H_Bnzy_⋯O (Bnzdzl = benzo­diazole and Bnzy = benz­yl) hydrogen bonds (Table 1 ▸), enclosing 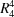
(27) ring motifs, into a network consisting of rectangular layers parallel to the *bc* plane (Fig. 2 ▸), which stack along the *a*-axis direction being associated through C—H⋯π (ring) inter­actions (Fig. 3 ▸).

## Hirshfeld surface analysis   

**Figure 4:**
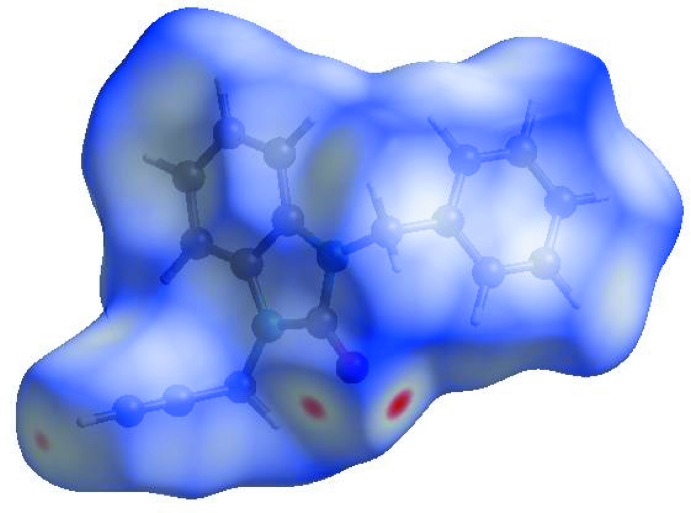
View of the three-dimensional Hirshfeld surface of the title compound plotted over *d*_norm_ in the range −0.1150 to 1.2702 a.u.

**Figure 5:**
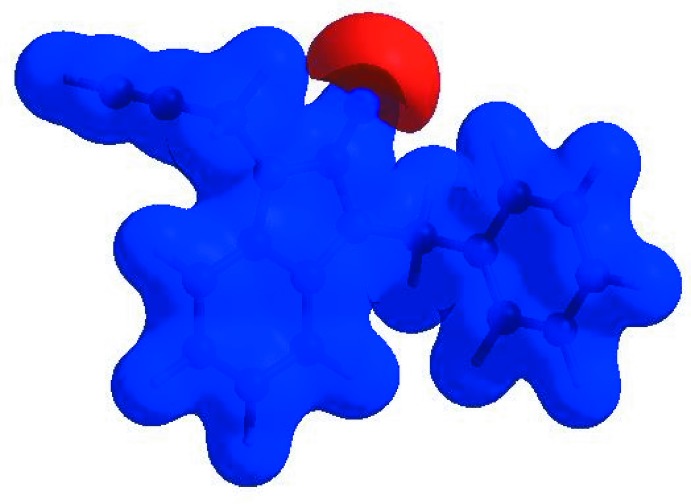
View of the three-dimensional Hirshfeld surface of the title compound plotted over electrostatic potential energy in the range −0.0500 to 0.0500 a.u. using the STO-3 G basis set at the Hartree–Fock level of theory hydrogen-bond donors and acceptors are shown as blue and red regions around the atoms corresponding to positive and negative potentials, respectively.

**Figure 6:**
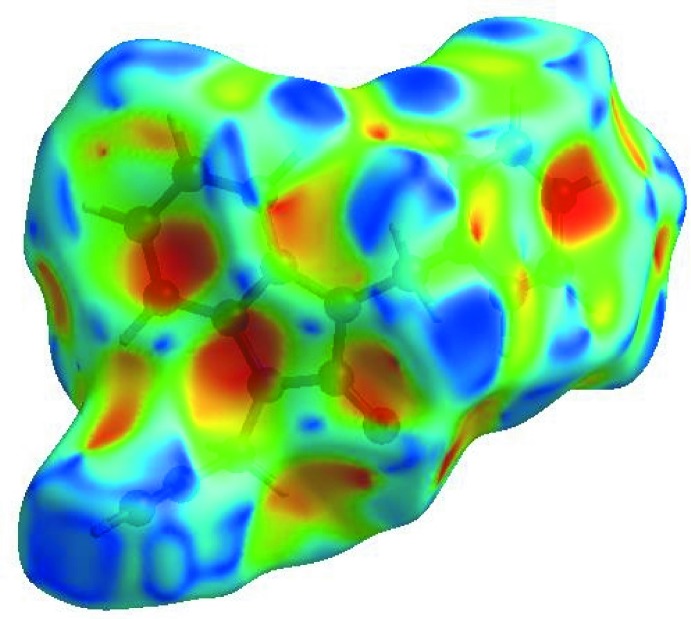
Hirshfeld surface of the title compound plotted over shape-index.

**Figure 7:**
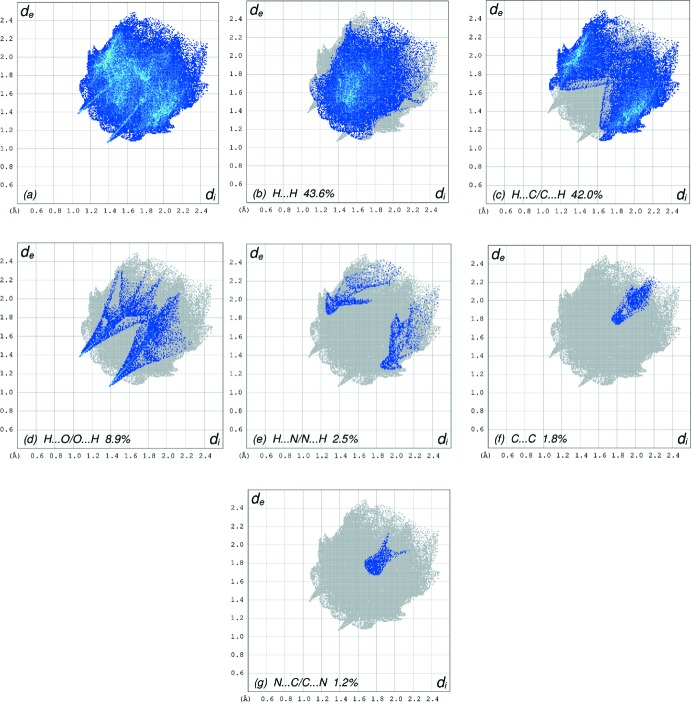
The full two-dimensional fingerprint plots for the title compound, showing (*a*) all inter­actions, and those delineated into (*b*) H⋯H, (*c*) H⋯C/C⋯H, (*d*) H⋯O/O⋯H, (*e*) H⋯N/N⋯H, (*f*) C⋯C and (*g*) N⋯C/C⋯N inter­actions. The *d*_i_ and *d*_e_ values are the closest inter­nal and external distances (in Å) from given points on the Hirshfeld surface contacts.

In order to visualize the inter­molecular inter­actions in the crystal of the title compound, a Hirshfeld surface (HS) analysis (Hirshfeld, 1977 ▸; Spackman & Jayatilaka, 2009 ▸) was carried out using *CrystalExplorer17.5* (Turner *et al.*, 2017 ▸). In the HS plotted over *d*_norm_ (Fig. 4 ▸), the white surface indicates contacts with distances equal to the sum of van der Waals radii, and the red and blue colours indicate distances shorter (in close contact) or longer (distinct contact) than the van der Waals radii, respectively (Venkatesan *et al.*, 2016 ▸). The bright-red spot appearing near O1 indicates its role as acceptor in the dominant C—H⋯O hydrogen bonds. Hydrogen-bond donors and acceptors appear, respectively, as blue and red regions corresponding to positive and negative potentials on the HS mapped over electrostatic potential (Spackman *et al.*, 2008 ▸; Jayatilaka *et al.*, 2005 ▸) shown in Fig. 5 ▸. The shape-index of the HS is a tool to visualize the π–π stacking by the presence of adjacent red and blue triangles; if there are no adjacent red and/or blue triangles, then there are no π–π inter­actions. Fig. 6 ▸ clearly suggests that there are no π–π inter­actions present. The overall two-dimensional fingerprint plot, Fig. 7 ▸(*a*), and those delineated into H⋯H, H⋯C/C⋯H, H⋯O/O⋯H, H⋯N/N⋯H, C⋯C and N⋯C/C⋯N contacts (McKinnon *et al.*, 2007 ▸) are illustrated in Fig. 7 ▸(*b*)–(*g*), respectively, together with their relative contributions to the Hirshfeld surface. The most important inter­action type is H⋯H, contributing 43.6% to the overall crystal packing, which is reflected in Fig. 7 ▸(*b*) as widely scattered points of high density due to the large hydrogen content of the mol­ecule and also due to the short H⋯H contacts (Table 2 ▸). In the presence of C—H⋯π inter­actions, the pair of widely scattered points of wings in the fingerprint plot delineated into H⋯C/C⋯H contacts (42.0% contribution to the HS) have a nearly symmetrical distribution of points, Fig. 7 ▸(*c*), with the tips at *d*_e_ + *d*_i_ ∼2.72 Å. The pair of characteristic wings in the fingerprint plot delineated into H⋯O/O⋯H contacts (8.9% contribution), Fig. 7 ▸(*d*), arises from the C—H⋯O hydrogen bonds (Table 1 ▸) as well as from the H⋯O/O⋯H contacts (Table 3 ▸) and has a pair of spikes with the tips at *d*_e_ + *d*_i_ = 2.43 Å. The pair of characteristic wings resulting in the fingerprint plot delineated into H ⋯ N/N ⋯ H contacts [Fig. 7 ▸(*e*), 2.5% contribution] has a pair of spikes with the tips at *d*_e_ + *d*_i_ = 3.12 Å. Finally, the wide spike with the tip at *d*_e_ = *d*_i_ = 1.77 Å in Fig. 7 ▸(*f*) is due to the C⋯C contacts (Table 3 ▸).

**Figure 8:**
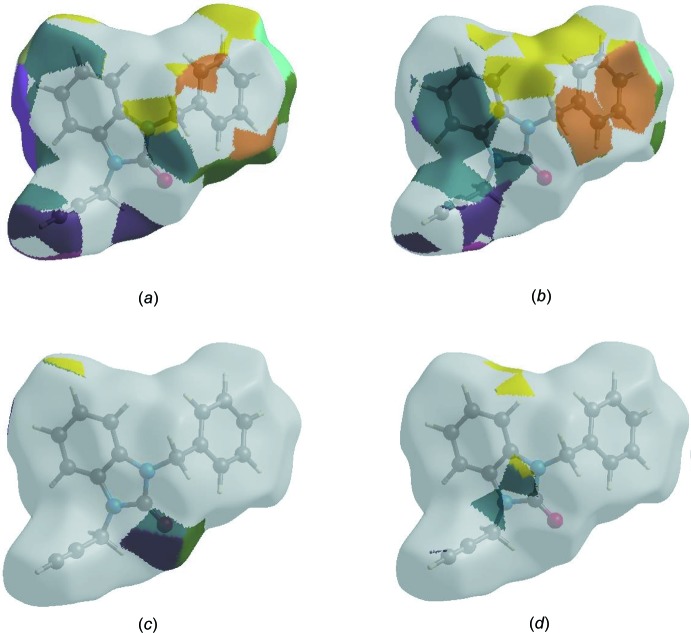
The Hirshfeld surface representations with the function *d*_norm_ plotted onto the surface for (*a*) H⋯H, (*b*) H⋯C/C⋯H, (*c*) H⋯O/O⋯H and (*d*) H⋯O/O⋯H inter­actions.

The Hirshfeld surface representations with the function *d*_norm_ plotted onto the surface are shown for the H⋯H, H⋯C/C⋯H, H⋯O/O⋯H and H⋯O/O⋯H inter­actions in Fig. 8 ▸(*a*)–(*d*), respectively.

The Hirshfeld surface analysis confirms the importance of H-atom contacts in establishing the packing. The large number of H⋯H, H⋯O/O⋯H and H⋯C/C⋯H inter­actions suggest that van der Waals inter­actions and hydrogen bonding play the major roles in the crystal packing (Hathwar *et al.*, 2015 ▸).

## Synthesis and crystallization   

To a solution of 1-(prop-2-yn­yl)-1*H*-benzoimidazol-2(3*H*)-one (3.42 mmol), benzyl chloride (6.81 mmol) and potassium carbonate (6.42 mmol) in DMF (15 ml) was added a catalytic amount of tetra-*n*-butyl­ammonium bromide (0.37 mmol) and the mixture was stirred for 24 h. The solid material was removed by filtration and the solvent evaporated under vacuum. The solid product was purified by recrystallization from ethanol to afford colourless crystals in 76% yield.

## Refinement   

Crystal data, data collection and structure refinement details are summarized in Table 3 ▸. Hydrogen atoms were located in a difference-Fourier map and freely refined.

## Supplementary Material

Crystal structure: contains datablock(s) I, global. DOI: 10.1107/S2056989018016298/xu5952sup1.cif

Structure factors: contains datablock(s) I. DOI: 10.1107/S2056989018016298/xu5952Isup2.hkl

Supporting information file. DOI: 10.1107/S2056989018016298/xu5952Isup3.cdx

CCDC reference: 1879758

Additional supporting information:  crystallographic information; 3D view; checkCIF report

## Figures and Tables

**Table 1:** Hydrogen-bond geometry (Å, °) *Cg*2 is the centroid of the C1–C6 benzene ring.

*D*—H⋯*A*	*D*—H	H⋯*A*	*D*⋯*A*	*D*—H⋯*A*
C3—H3⋯O1^iii^	0.982 (18)	2.542 (18)	3.4997 (18)	165.1 (14)
C16—H16⋯O1^vi^	0.994 (18)	2.568 (18)	3.468 (2)	150.6 (14)
C17—H17⋯*Cg*2^viii^	1.00 (2)	2.831 (18)	3.6964 (17)	144.6 (15)

Symmetry codes: (iii) 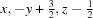; (vi) 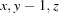; (viii) 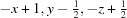.

**Table 2:** Selected interatomic distances (Å)

O1⋯H8*B*	2.492 (18)	C5⋯H11*A*	2.897 (16)
O1⋯H11*B*	2.607 (16)	C5⋯H10^vi^	2.93 (3)
O1⋯H13	2.760 (18)	C8⋯H2	2.947 (16)
O1⋯H16^i^	2.568 (18)	C10⋯H8*A* ^ii^	2.874 (18)
C2⋯C9	3.5265 (19)	C10⋯H14^iii^	2.95 (2)
C7⋯C13	3.5805 (19)	C10⋯H11*A* ^vii^	2.895 (17)
C9⋯C1^ii^	3.5236 (18)	C11⋯H5	2.998 (16)
C10⋯N2^ii^	3.4291 (19)	C13⋯H14^v^	2.964 (17)
C10⋯C8^ii^	3.385 (2)	H3⋯O1^iii^	2.542 (18)
C10⋯C14^iii^	3.512 (3)	H5⋯H11*A*	2.46 (2)
C11⋯C13^iv^	3.495 (2)	H11*A*⋯H17	2.38 (2)
C14⋯C14^v^	3.543 (2)	H11*B*⋯H13	2.45 (2)
C4⋯H10^vi^	2.84 (3)		

Symmetry codes: (i) 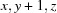; (ii) 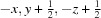; (iii) 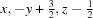; (iv) 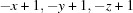; (v) 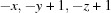; (vi) 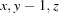; (vii) 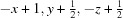.

**Table 3:** Experimental details

Crystal data	
Chemical formula	C_17_H_14_N_2_O
*M*_r_	262.30
Crystal system, space group	Monoclinic, *P*2_1_/*c*
Temperature (K)	298
*a*, *b*, *c* (Å)	8.3567 (2), 9.2040 (2), 17.7868 (4)
β (°)	94.559 (1)
*V* (Å^3^)	1363.74 (5)
*Z*	4
Radiation type	Cu *K*α
μ (mm^−1^)	0.64
Crystal size (mm)	0.23 × 0.20 × 0.19

Data collection	
Diffractometer	Bruker D8 VENTURE PHOTON 100 CMOS
Absorption correction	Multi-scan (*SADABS*; Krause *et al.*, 2015 ▸)
*T*_min_, *T*_max_	0.86, 0.89
No. of measured, independent and observed [*I* > 2σ(*I*)] reflections	13551, 2778, 2433
*R*_int_	0.032
(sin θ/λ)_max_ (Å^−1^)	0.625

Refinement	
*R*[*F* ^2^ > 2σ(*F* ^2^)], *wR*(*F* ^2^), *S*	0.038, 0.108, 1.05
No. of reflections	2778
No. of parameters	238
H-atom treatment	All H-atom parameters refined
Δρ_max_, Δρ_min_ (e Å^−3^)	0.15, −0.12

Computer programs: *APEX3* and *SAINT* (Bruker, 2016 ▸), *SHELXT* (Sheldrick, 2015*a* ▸), *SHELXL2018/1* (Sheldrick, 2015*b* ▸), *Mercury* (Macrae, *et al.*, 2008 ▸) and *SHELXTL* (Sheldrick, 2008 ▸).

## supplementary crystallographic information

### Crystal data

**Table:**

C_17_H_14_N_2_O	*F*(000) = 552
*M**_r_* = 262.30	*D*_x_ = 1.278 Mg m^−^^3^
Monoclinic, *P*2_1_/*c*	Cu *K*α radiation, λ = 1.54178 Å
*a* = 8.3567 (2) Å	Cell parameters from 9908 reflections
*b* = 9.2040 (2) Å	θ = 2.5–74.9°
*c* = 17.7868 (4) Å	µ = 0.64 mm^−^^1^
β = 94.559 (1)°	*T* = 298 K
*V* = 1363.74 (5) Å^3^	Block, colourless
*Z* = 4	0.23 × 0.20 × 0.19 mm

### Data collection

**Table:**

Bruker D8 VENTURE PHOTON 100 CMOS diffractometer	2433 reflections with *I* > 2σ(*I*)
Radiation source: INCOATEC IµS micro-focus source	*R*_int_ = 0.032
ω scans	θ_max_ = 74.4°, θ_min_ = 5.0°
Absorption correction: multi-scan (*SADABS*; Krause *et al.*, 2015)	*h* = −10→10
*T*_min_ = 0.86, *T*_max_ = 0.89	*k* = −11→11
13551 measured reflections	*l* = −22→21
2778 independent reflections	

### Refinement

**Table:**

Refinement on *F*^2^	Secondary atom site location: difference Fourier map
Least-squares matrix: full	Hydrogen site location: difference Fourier map
*R*[*F*^2^ > 2σ(*F*^2^)] = 0.038	All H-atom parameters refined
*wR*(*F*^2^) = 0.108	*w* = 1/[σ^2^(*F*_o_^2^) + (0.0559*P*)^2^ + 0.1595*P*] where *P* = (*F*_o_^2^ + 2*F*_c_^2^)/3
*S* = 1.05	(Δ/σ)_max_ < 0.001
2778 reflections	Δρ_max_ = 0.15 e Å^−^^3^
238 parameters	Δρ_min_ = −0.12 e Å^−^^3^
0 restraints	Extinction correction: *SHELXL-2018/1* (Sheldrick, 2015*b*), Fc^*^=kFc[1+0.001xFc^2^λ^3^/sin(2θ)]^-1/4^
Primary atom site location: structure-invariant direct methods	Extinction coefficient: 0.0123 (10)

### Special details

**Table:**

Geometry. All esds (except the esd in the dihedral angle between two l.s. planes) are estimated using the full covariance matrix. The cell esds are taken into account individually in the estimation of esds in distances, angles and torsion angles; correlations between esds in cell parameters are only used when they are defined by crystal symmetry. An approximate (isotropic) treatment of cell esds is used for estimating esds involving l.s. planes.
Refinement. Refinement of F^2^ against ALL reflections. The weighted R-factor wR and goodness of fit S are based on F^2^, conventional R-factors R are based on F, with F set to zero for negative F^2^. The threshold expression of F^2^ > 2sigma(F^2^) is used only for calculating R-factors(gt) etc. and is not relevant to the choice of reflections for refinement. R-factors based on F^2^ are statistically about twice as large as those based on F, and R- factors based on ALL data will be even larger.

### Fractional atomic coordinates and isotropic or equivalent isotropic displacement parameters (Å^2^)

**Table:**

	*x*	*y*	*z*	*U*_iso_*/*U*_eq_	
O1	0.22681 (15)	0.83445 (11)	0.40629 (6)	0.0792 (3)	
N1	0.34376 (12)	0.66295 (10)	0.33147 (5)	0.0533 (3)	
N2	0.17477 (12)	0.82074 (10)	0.27629 (6)	0.0543 (3)	
C1	0.22401 (13)	0.73060 (12)	0.22001 (6)	0.0491 (3)	
C2	0.18494 (17)	0.72757 (15)	0.14354 (7)	0.0629 (3)	
H2	0.1121 (19)	0.7990 (18)	0.1214 (9)	0.075 (4)*	
C3	0.2547 (2)	0.62075 (18)	0.10228 (8)	0.0734 (4)	
H3	0.234 (2)	0.617 (2)	0.0472 (10)	0.090 (5)*	
C4	0.3609 (2)	0.52123 (16)	0.13680 (8)	0.0703 (4)	
H4	0.414 (2)	0.4500 (18)	0.1060 (9)	0.077 (4)*	
C5	0.40140 (16)	0.52408 (14)	0.21399 (8)	0.0590 (3)	
H5	0.476 (2)	0.4532 (18)	0.2392 (9)	0.077 (4)*	
C6	0.33092 (13)	0.63007 (12)	0.25513 (6)	0.0478 (3)	
C7	0.24590 (16)	0.77932 (13)	0.34550 (7)	0.0555 (3)	
C8	0.06278 (18)	0.94116 (15)	0.26623 (10)	0.0675 (4)	
H8A	−0.034 (2)	0.908 (2)	0.2331 (11)	0.095 (6)*	
H8B	0.030 (2)	0.965 (2)	0.3179 (11)	0.089 (5)*	
C9	0.13423 (17)	1.06640 (14)	0.23111 (8)	0.0633 (3)	
C10	0.1922 (2)	1.16568 (17)	0.20289 (11)	0.0854 (5)	
H10	0.236 (3)	1.249 (3)	0.1798 (13)	0.132 (8)*	
C11	0.43747 (17)	0.58361 (15)	0.39115 (8)	0.0600 (3)	
H11A	0.539 (2)	0.5429 (18)	0.3684 (9)	0.080 (5)*	
H11B	0.4649 (19)	0.6538 (18)	0.4327 (10)	0.080 (5)*	
C12	0.34639 (14)	0.45907 (12)	0.42256 (6)	0.0512 (3)	
C13	0.23968 (18)	0.48403 (17)	0.47628 (8)	0.0675 (4)	
H13	0.223 (2)	0.587 (2)	0.4938 (9)	0.087 (5)*	
C14	0.1570 (2)	0.3702 (2)	0.50568 (10)	0.0819 (5)	
H14	0.086 (2)	0.387 (2)	0.5429 (12)	0.107 (6)*	
C15	0.1808 (2)	0.2308 (2)	0.48218 (11)	0.0830 (5)	
H15	0.123 (2)	0.154 (2)	0.5025 (10)	0.095 (6)*	
C16	0.2874 (3)	0.20500 (18)	0.42939 (11)	0.0870 (5)	
H16	0.306 (2)	0.104 (2)	0.4124 (11)	0.106 (6)*	
C17	0.3696 (2)	0.31841 (15)	0.39958 (9)	0.0701 (4)	
H17	0.446 (2)	0.299 (2)	0.3599 (12)	0.103 (6)*	

### Atomic displacement parameters (Å^2^)

**Table:**

	*U*^11^	*U*^22^	*U*^33^	*U*^12^	*U*^13^	*U*^23^
O1	0.1247 (9)	0.0571 (6)	0.0569 (5)	0.0079 (5)	0.0134 (5)	−0.0057 (4)
N1	0.0663 (6)	0.0445 (5)	0.0475 (5)	0.0034 (4)	−0.0042 (4)	0.0037 (4)
N2	0.0612 (6)	0.0435 (5)	0.0583 (6)	0.0059 (4)	0.0049 (4)	0.0066 (4)
C1	0.0519 (6)	0.0436 (6)	0.0512 (6)	−0.0061 (4)	0.0006 (4)	0.0055 (4)
C2	0.0717 (8)	0.0593 (7)	0.0557 (7)	−0.0085 (6)	−0.0081 (6)	0.0106 (6)
C3	0.1009 (11)	0.0707 (9)	0.0478 (7)	−0.0184 (8)	0.0008 (7)	0.0001 (6)
C4	0.0940 (10)	0.0571 (8)	0.0619 (8)	−0.0090 (7)	0.0197 (7)	−0.0083 (6)
C5	0.0661 (7)	0.0469 (6)	0.0645 (7)	0.0002 (5)	0.0090 (6)	0.0013 (5)
C6	0.0524 (6)	0.0413 (5)	0.0493 (6)	−0.0050 (4)	0.0014 (4)	0.0035 (4)
C7	0.0727 (8)	0.0420 (6)	0.0520 (6)	−0.0020 (5)	0.0054 (5)	0.0027 (5)
C8	0.0649 (8)	0.0524 (7)	0.0868 (10)	0.0123 (6)	0.0158 (7)	0.0174 (7)
C9	0.0715 (8)	0.0472 (6)	0.0723 (8)	0.0125 (6)	0.0130 (6)	0.0067 (6)
C10	0.1019 (12)	0.0519 (8)	0.1070 (13)	0.0087 (8)	0.0373 (10)	0.0107 (8)
C11	0.0641 (7)	0.0574 (7)	0.0560 (7)	−0.0024 (6)	−0.0113 (6)	0.0091 (6)
C12	0.0549 (6)	0.0490 (6)	0.0478 (6)	0.0059 (5)	−0.0082 (5)	0.0057 (5)
C13	0.0748 (8)	0.0627 (8)	0.0652 (8)	0.0149 (7)	0.0066 (6)	0.0057 (6)
C14	0.0666 (8)	0.0971 (12)	0.0833 (10)	0.0159 (8)	0.0131 (8)	0.0295 (9)
C15	0.0703 (9)	0.0791 (11)	0.0967 (12)	−0.0107 (8)	−0.0120 (8)	0.0368 (9)
C16	0.1131 (14)	0.0506 (8)	0.0956 (12)	−0.0019 (8)	−0.0018 (10)	0.0065 (8)
C17	0.0896 (10)	0.0526 (7)	0.0687 (8)	0.0097 (7)	0.0099 (7)	0.0036 (6)

### Geometric parameters (Å, º)

**Table:**

O1—C7	1.2163 (15)	C8—H8A	1.01 (2)
N1—C7	1.3823 (16)	C8—H8B	1.004 (18)
N1—C6	1.3869 (15)	C9—C10	1.166 (2)
N1—C11	1.4632 (15)	C10—H10	0.95 (2)
N2—C7	1.3775 (16)	C11—C12	1.5076 (17)
N2—C1	1.3875 (15)	C11—H11A	1.042 (17)
N2—C8	1.4525 (16)	C11—H11B	0.995 (18)
C1—C2	1.3737 (17)	C12—C17	1.3761 (18)
C1—C6	1.3985 (16)	C12—C13	1.3771 (19)
C2—C3	1.383 (2)	C13—C14	1.381 (2)
C2—H2	0.959 (17)	C13—H13	1.008 (19)
C3—C4	1.384 (2)	C14—C15	1.369 (3)
C3—H3	0.982 (18)	C14—H14	0.94 (2)
C4—C5	1.388 (2)	C15—C16	1.366 (3)
C4—H4	0.983 (17)	C15—H15	0.94 (2)
C5—C6	1.3793 (17)	C16—C17	1.379 (2)
C5—H5	0.986 (17)	C16—H16	0.99 (2)
C8—C9	1.4610 (19)	C17—H17	1.00 (2)
			
O1···H8B	2.492 (18)	C5···H11A	2.897 (16)
O1···H11B	2.607 (16)	C5···H10^vi^	2.93 (3)
O1···H13	2.760 (18)	C8···H2	2.947 (16)
O1···H16^i^	2.568 (18)	C10···H8A^ii^	2.874 (18)
C2···C9	3.5265 (19)	C10···H14^iii^	2.95 (2)
C7···C13	3.5805 (19)	C10···H11A^vii^	2.895 (17)
C9···C1^ii^	3.5236 (18)	C11···H5	2.998 (16)
C10···N2^ii^	3.4291 (19)	C13···H14^v^	2.964 (17)
C10···C8^ii^	3.385 (2)	H3···O1^iii^	2.542 (18)
C10···C14^iii^	3.512 (3)	H5···H11A	2.46 (2)
C11···C13^iv^	3.495 (2)	H11A···H17	2.38 (2)
C14···C14^v^	3.543 (2)	H11B···H13	2.45 (2)
C4···H10^vi^	2.84 (3)		
			
C7—N1—C6	110.18 (9)	N2—C8—H8B	105.8 (10)
C7—N1—C11	123.09 (10)	C9—C8—H8B	111.9 (11)
C6—N1—C11	126.62 (10)	H8A—C8—H8B	109.7 (15)
C7—N2—C1	110.34 (9)	C10—C9—C8	179.48 (16)
C7—N2—C8	123.32 (11)	C9—C10—H10	178.1 (14)
C1—N2—C8	126.34 (11)	N1—C11—C12	113.05 (10)
C2—C1—N2	131.74 (11)	N1—C11—H11A	107.8 (9)
C2—C1—C6	121.46 (11)	C12—C11—H11A	108.8 (9)
N2—C1—C6	106.80 (10)	N1—C11—H11B	107.1 (10)
C1—C2—C3	117.56 (13)	C12—C11—H11B	108.2 (9)
C1—C2—H2	119.0 (10)	H11A—C11—H11B	112.0 (13)
C3—C2—H2	123.4 (10)	C17—C12—C13	118.52 (13)
C2—C3—C4	121.16 (13)	C17—C12—C11	121.19 (12)
C2—C3—H3	120.6 (11)	C13—C12—C11	120.28 (12)
C4—C3—H3	118.2 (11)	C12—C13—C14	120.50 (15)
C3—C4—C5	121.56 (14)	C12—C13—H13	119.1 (10)
C3—C4—H4	119.7 (10)	C14—C13—H13	120.4 (10)
C5—C4—H4	118.6 (10)	C15—C14—C13	120.41 (16)
C6—C5—C4	117.22 (13)	C15—C14—H14	119.1 (14)
C6—C5—H5	120.5 (10)	C13—C14—H14	120.5 (14)
C4—C5—H5	122.3 (10)	C16—C15—C14	119.46 (16)
C5—C6—N1	132.12 (11)	C16—C15—H15	120.9 (11)
C5—C6—C1	121.03 (11)	C14—C15—H15	119.6 (11)
N1—C6—C1	106.85 (10)	C15—C16—C17	120.33 (16)
O1—C7—N2	126.95 (12)	C15—C16—H16	119.8 (12)
O1—C7—N1	127.23 (12)	C17—C16—H16	119.8 (12)
N2—C7—N1	105.82 (10)	C12—C17—C16	120.78 (15)
N2—C8—C9	111.93 (11)	C12—C17—H17	119.1 (11)
N2—C8—H8A	108.6 (11)	C16—C17—H17	120.1 (11)
C9—C8—H8A	108.8 (11)		
			
C7—N2—C1—C2	179.03 (13)	C1—N2—C7—N1	1.23 (13)
C8—N2—C1—C2	−0.1 (2)	C8—N2—C7—N1	−179.61 (11)
C7—N2—C1—C6	−0.58 (13)	C6—N1—C7—O1	178.85 (13)
C8—N2—C1—C6	−179.71 (11)	C11—N1—C7—O1	2.4 (2)
N2—C1—C2—C3	−179.26 (12)	C6—N1—C7—N2	−1.44 (13)
C6—C1—C2—C3	0.29 (18)	C11—N1—C7—N2	−177.93 (11)
C1—C2—C3—C4	−0.4 (2)	C7—N2—C8—C9	107.51 (15)
C2—C3—C4—C5	0.2 (2)	C1—N2—C8—C9	−73.46 (18)
C3—C4—C5—C6	0.2 (2)	C7—N1—C11—C12	88.90 (15)
C4—C5—C6—N1	179.75 (12)	C6—N1—C11—C12	−87.00 (15)
C4—C5—C6—C1	−0.37 (18)	N1—C11—C12—C17	99.61 (15)
C7—N1—C6—C5	−179.00 (12)	N1—C11—C12—C13	−81.60 (15)
C11—N1—C6—C5	−2.7 (2)	C17—C12—C13—C14	−0.6 (2)
C7—N1—C6—C1	1.11 (13)	C11—C12—C13—C14	−179.46 (13)
C11—N1—C6—C1	177.44 (11)	C12—C13—C14—C15	0.4 (2)
C2—C1—C6—C5	0.12 (17)	C13—C14—C15—C16	0.1 (3)
N2—C1—C6—C5	179.78 (10)	C14—C15—C16—C17	−0.5 (3)
C2—C1—C6—N1	−179.98 (11)	C13—C12—C17—C16	0.3 (2)
N2—C1—C6—N1	−0.32 (12)	C11—C12—C17—C16	179.09 (14)
C1—N2—C7—O1	−179.06 (13)	C15—C16—C17—C12	0.3 (3)
C8—N2—C7—O1	0.1 (2)		

Symmetry codes: (i) *x*, *y*+1, *z*; (ii) −*x*, *y*+1/2, −*z*+1/2; (iii) *x*, −*y*+3/2, *z*−1/2; (iv) −*x*+1, −*y*+1, −*z*+1; (v) −*x*, −*y*+1, −*z*+1; (vi) *x*, *y*−1, *z*; (vii) −*x*+1, *y*+1/2, −*z*+1/2.

### Hydrogen-bond geometry (Å, º)

Cg2 is the centroid of the C1–C6 benzene ring.

**Table:**

*D*—H···*A*	*D*—H	H···*A*	*D*···*A*	*D*—H···*A*
C3—H3···O1^iii^	0.982 (18)	2.542 (18)	3.4997 (18)	165.1 (14)
C16—H16···O1^vi^	0.994 (18)	2.568 (18)	3.468 (2)	150.6 (14)
C17—H17···*Cg*2^viii^	1.00 (2)	2.831 (18)	3.6964 (17)	144.6 (15)

Symmetry codes: (iii) *x*, −*y*+3/2, *z*−1/2; (vi) *x*, *y*−1, *z*; (viii) −*x*+1, *y*−1/2, −*z*+1/2.
